# Joint network analysis of non-motor symptoms and quality of life in patients with Parkinson’s disease

**DOI:** 10.3389/fnagi.2026.1939150

**Published:** 2026-09-03

**Authors:** CaiNi Wu, XueYing Wang, JiaJia Li, ShaoHua Yang, Xue Cong, Yi Jin

**Affiliations:** 1 Tianjin University of Traditional Chinese Medicine, Tianjin, China; 2 Huanhu Hospital Affiliated to Tianjin Medical University, Tianjin, China; 3 Department of Nursing, Beijing Shijitan Hospital, Capital Medical University, Beijing, China; 4 Discipline Inspection Office of Huanhu Hospital Affiliated to Tianjin Medical University, Tianjin, China

**Keywords:** bridge centrality, network analysis, non-motor symptoms, Parkinson’s disease, quality of life

## Abstract

**Background:**

Non-motor symptoms (NMS) are highly prevalent in Parkinson’s disease (PD) and are major determinants of declining quality of life (QoL), yet traditional statistical approaches are insufficient to capture the complex interactions between NMS and QoL. Network analysis offers a promising alternative for exploring these interactions.

**Objective:**

To construct a joint network of non-motor symptoms (NMS) and quality of life (QoL) in patients with Parkinson’s disease (PD), identify core and bridge nodes, and inform precision nursing interventions.

**Methods:**

A convenience sample of 342 PD patients was recruited between January and June 2026 from the PD specialty outpatient clinic and neurology inpatient wards of a tertiary specialized hospital in Tianjin, and investigated using the Non-Motor Symptoms Scale (NMSS) and the Parkinson’s Disease Questionnaire-39 (PDQ-39). A joint network model was constructed via network analysis, and network structures were compared between early- and mid-to-late-stage patients.

**Results:**

NMSS6 gastrointestinal symptoms (Str = 1.027), PDQ5 social support (Str = 0.995), and PDQ6 cognition (Str = 0.992) were the core nodes of the joint network based on strength centrality. PDQ6 cognition (Bridge Str = 0.326) and NMSS3 mood/cognition (Bridge Str = 0.260) were the key bridge symptoms linking NMS and QoL. Mobility and social support showed the highest expected influence (EI = 0.960 and 0.939, respectively). The overall network structure differed significantly between early-stage and mid-to-late-stage patients (*p* = 0.004), whereas global network strength did not; after correction for multiple comparisons.

**Conclusion:**

Complex associations exist between NMS and QoL in PD patients and are reorganized across disease stages; gastrointestinal symptoms, cognition, and social support represent candidate targets for future longitudinal and interventional research.

## Introduction

Parkinson’s disease (PD) is the second most common neurodegenerative disorder worldwide, with more than 11.77 million patients currently affected globally. As population aging accelerates, the prevalence of PD among individuals aged 60 years and above in China is approximately 1.37%, with more than 5 million patients—accounting for roughly half of the global total—resulting in an increasingly heavy disease burden (Wu et al., 2026). The clinical presentation of PD is complex and heterogeneous: in addition to motor symptoms such as resting tremor, rigidity, bradykinesia, and postural instability, patients also experience a wide range of non-motor symptoms (NMS), including sleep disturbances, cognitive decline, depression and anxiety, and autonomic dysfunction (Leite Silva et al., 2023). NMS occur throughout the entire disease course, with a high prevalence but low recognition rate, and exert a sustained negative impact on patients’ daily functioning, social participation, and psychological well-being. As such, NMS are a major determinant of declining quality of life (QoL) in patients with PD, with an impact that may even exceed that of motor symptoms (Bloem et al., 2021; Kuhlman et al., 2019).

However, most existing studies have focused on single symptoms such as depression, sleep disturbance, or cognitive impairment (Kwok et al., 2025; Stefani and HöGL, 2020; Oikonomou et al., 2026), or have relied on traditional statistical approaches such as correlation and regression analysis to examine the relationship between NMS and QoL (Hamedani et al., 2023; Cassidy et al., 2022; Bock et al., 2022), which are insufficient to reveal the complex interactions among different symptoms and QoL dimensions. Moreover, the burden of NMS and QoL differs significantly between patients in early and mid-to-late disease stages (Kumar et al., 2022), yet few studies have examined similarities and differences in their association patterns from a disease-stage perspective. Network analysis is an emerging statistical approach that has recently been applied to symptom research in chronic diseases; it integrates NMS and QoL dimensions into a single network and reveals complex interactions among variables by identifying core nodes, bridge nodes, expected influence, and network structural characteristics (Yang et al., 2022; Jones et al., 2021). Several studies have applied network analysis to NMS and QoL in PD: Heimrich et al. (2023b) examined item-level associations between the NMSS and PDQ-39 in 689 patients from the COPPADIS cohort and subsequently examined longitudinal changes in item-level NMSS networks; Schönenberg et al. (2023) investigated the internal item structure of the PDQ-39; and Deng et al. (2025) examined a symptom-QoL network in a cohort of Chinese patients with early-to-mid-stage PD. These studies provide valuable item-level insights but, to our knowledge, have not modeled all NMSS and PDQ-39 domains together within a single joint network. Whereas item-level analyses capture symptom-specific relationships, total scores obscure associations between particular NMS and QoL domains. Modeling all nine NMSS and eight PDQ-39 domains together therefore provides a complementary and clinically interpretable map of within- and cross-scale associations. Whether this domain-level architecture differs between H-Y stage-defined groups also remains unclear. We therefore constructed a joint domain-level network, estimated cross-scale bridge centrality, and exploratorily compared early-stage and mid-to-late-stage groups.

## Methods

### Participants

A convenience sampling method was used to recruit patients with Parkinson’s disease (PD) from the PD specialty outpatient clinic and neurology inpatient wards of a tertiary specialized hospital in Tianjin, China, between January and June 2026.

### Selection criteria

(I) a diagnosis of idiopathic PD according to the latest Diagnostic Criteria for Parkinson’s Disease in China (2016 edition) (Parkinson’s Disease and Movement Disorders Group of Neurology Branch, Chinese Medical Association; Parkinson’s Disease and Movement Disorders Professional Committee of Neurologist Branch, Chinese Medical Doctor Association, 2016), confirmed by a movement-disorder-trained neurologist at the study hospital; (II) age ≥18 years; (III) clear consciousness with sufficient communication and comprehension ability to complete the questionnaires, confirmed by clinicians through a brief bedside assessment before enrollment, which also confirmed the absence of clinically apparent dementia; and (IV) provision of informed consent and voluntary participation. The exclusion criteria were: (I) comorbid severe psychiatric disorders; (II) comorbid severe cardiac, hepatic, or renal insufficiency; (III) comorbid Parkinsonism or other neurological disorders; and (IV) severe hearing, visual, or language impairment precluding completion of the survey. *These criteria applied only to severe conditions*; *patients with stable chronic comorbidities remained eligible for inclusion*.

### Sample size

According to the sample size requirements for network analysis (Papachristou et al., 2019), a network containing n nodes requires n + n × (n-1)/2 parameters, and the sample size should be at least equal to the number of parameters to ensure reliable parameter estimation. We clarified that this rule of thumb represents only a preliminary minimum and does not guarantee the accuracy or stability of an EBICglasso network. Therefore, we additionally assessed edge-weight accuracy using bootstrap confidence intervals and centrality stability using case-dropping subset bootstrap analysis (Epskamp et al., 2018) (see Statistical Analysis and Results). As the present study included 17 nodes, and allowing for a 20% rate of invalid questionnaires, a minimum of 192 participants was required. A total of 342 participants were ultimately enrolled. The stage-stratified analysis is now explicitly identified as exploratory because of the subgroup sample sizes and the differences in age and disease duration between the two groups. This study was approved by the Medical Ethics Committee of the study hospital (approval No. 2025–283).

### Demographic and clinical characteristics questionnaire

A self-designed questionnaire was used to collect sociodemographic and disease-related characteristics, including sex, age, Hoehn-Yahr (H-Y) stage, average monthly household income per capita, educational level, disease duration, marital status, presence of comorbid chronic diseases, and employment status.

Non-Motor Symptoms Scale (NMSS). The NMSS was developed by Chaudhuri et al. (2007) and later translated and validated in Chinese by Wang et al. (2009). It comprises 30 items across nine domains: cardiovascular (2 items), sleep/fatigue (4 items), mood/cognition (6 items), perceptual problems/hallucinations (3 items), attention/memory (3 items), gastrointestinal tract (3 items), urinary (3 items), sexual function (2 items), and miscellaneous (4 items). Each item is rated on two dimensions—frequency (1–4) and severity (0–3)—and the item score is calculated as the product of the two ratings, yielding a total score ranging from 0 to 360. Higher scores indicate a greater burden of non-motor symptoms. In this study, Cronbach’s α for the NMSS was 0.852.

### Parkinson’s Disease Questionnaire-39 (PDQ-39)

The PDQ-39 was developed by Parkinson’s Disease and Movement Disorders Group of Neurology Branch, Chinese Medical Association; Parkinson’s Disease and Movement Disorders Professional Committee of Neurologist Branch, Chinese Medical Doctor Association (2016) and subsequently validated in Chinese by Luo et al. (2010). It consists of 39 items across eight domains: mobility (10 items), activities of daily living (6 items), emotional well-being (6 items), stigma (4 items), social support (3 items), cognition (4 items), communication (3 items), and bodily discomfort (3 items). Each item is scored from 0 to 4 (0 = never, 1 = occasionally, 2 = sometimes, 3 = often, 4 = always), and domain scores—calculated as the sum of item scores—are converted to a 0–100 percentage scale. Higher scores indicate poorer quality of life (QoL). To reflect the overall QoL burden, the sum of the eight percentage-scaled domain scores was also calculated as the PDQ-39 total score. In this study, Cronbach’s α for the PDQ-39 was 0.892.

### Data collection

Data were collected via face-to-face questionnaire surveys conducted by two trained investigators (full-time master’s students in nursing) who received standardized training prior to the study. Before each survey, the purpose, content, and precautions of the study were explained to participants, and data collection began only after informed consent was obtained. For participants with reading or writing difficulties, investigators used standardized instructions to orally administer the questionnaires and assist with completion, while avoiding leading questions.

### Quality control

Completed questionnaires were checked on-site by the investigators; missing or incomplete responses were supplemented immediately when possible, and questionnaires with a completion time of <5 min or patterned/repetitive responses were excluded. When a questionnaire could not be completed even with investigator assistance, it was excluded from the analytic sample. Of the 360 questionnaires distributed, 9 were excluded for a completion time <5 min, 5 for patterned/repetitive responses, and 4 for incomplete responses that could not be resolved through investigator assistance, together yielding the 342 valid questionnaires analyzed. All data were double-entered into the database, and logic checks and missing-value verification were performed to ensure data accuracy and completeness.

### Statistical analysis

Data were analyzed using SPSS software (version 27.0). The Kolmogorov–Smirnov test indicated that continuous variables were non-normally distributed; these variables were therefore expressed as median (P_25_, P_75_), and between-group comparisons were performed using the Mann–Whitney U test. Categorical variables were expressed as frequencies and percentages/rates.

Network analysis and visualization were performed using R software (version 4.6.0). A sparse, regularized partial correlation network was constructed using the qgraph package with the graphical least absolute shrinkage and selection operator combined with the extended Bayesian information criterion (EBICglasso, *γ* = 0.5), in which edge weights represent partial correlations between nodes. In the resulting network, solid and dashed edges denote positive and negative associations, respectively, and edge thickness is proportional to the strength of the association. Node strength centrality (Str) was calculated for each node, with higher values indicating greater importance of the node within the network. Betweenness and closeness centrality were not used to determine node importance, as recent methodological research has shown that these two indices display poor stability and questionable theoretical validity in psychological and symptom networks, and has recommended strength centrality as the primary index of node importance in such networks (Bringmann et al., 2019). Expected influence (EI) was also calculated to evaluate the overall influence of each node on the network. Bridge strength and bridge expected influence were using the bridge function in the networktools package to identify key bridge nodes linking non-motor symptoms (NMS) and quality of life (QoL); bridge strength sums the absolute weights and bridge expected influence the signed weights of the edges connecting a node to the other community.

Network stability and accuracy were assessed using the bootnet package. A centrality stability (CS) coefficient >0.5 was considered to indicate good stability, and a value >0.25 was considered acceptable. The 95% confidence intervals (CIs) of edge weights were estimated using a nonparametric bootstrap procedure (1,000 replications); narrower CIs indicate more accurate edge-weight estimates (Epskamp et al., 2018). Differences in network structure between early- and mid-to-late-stage patients were examined using the Network Comparison Test (NCT) package, which compares networks at three levels: global network structure invariance, global strength invariance, and edge-level (local) invariance. A two-sided α of 0.05 was considered statistically significant. The NCT was performed using 10,000 permutations with a fixed random seed. A two-sided α of 0.05 was considered statistically significant. *p* values from the 136 edge-level comparisons were adjusted using the Benjamini–Hochberg procedure to control the false discovery rate.

## Results

### General characteristics of patients with Parkinson’s disease

A total of 360 questionnaires were distributed, of which 342 valid questionnaires were returned, yielding an effective response rate of 95.0%. Among the 342 patients with Parkinson’s disease (PD), 183 (53.5%) were male and 159 (46.5%) were female, with a median age of 69.00 (64.00, 74.00) years. Regarding educational level, 167 patients (48.8%) had completed junior high school or below, 136 (39.8%) had a senior high school or technical secondary school education, and 39 (11.4%) had a junior college education or above. With respect to marital status, 327 patients (95.6%) were married and 15 (4.4%) were unmarried or divorced. Average monthly household income per capita was <3,000 CNY in 91 patients (26.6%), 3,000–5,000 CNY in 134 patients (39.2%), and >5,000 CNY in 117 patients (34.2%). Disease duration was <5 years in 110 patients (32.2%), 5–10 years in 148 patients (43.3%), and >10 years in 84 patients (24.6%). Regarding employment status, 17 patients (5.0%) were currently employed, 151 (44.2%) were retired, 97 (28.4%) were unemployed, and 77 (22.5%) were farmers. A total of 208 patients (60.8%) had at least one comorbid chronic disease, whereas 134 (39.2%) had none. According to Hoehn-Yahr (H-Y) staging, 151 patients were classified as early-stage (H-Y 1–2.5) and 191 as mid-to-late-stage (H-Y 3–5). No significant differences were observed between the two groups in sex, educational level, marital status, average monthly household income per capita, employment status, or presence of comorbid chronic diseases (all *p* > 0.05), whereas significant differences were found in age and disease duration (both *p* < 0.05) (Table 1).

**Table 1:** Comparison of general characteristics between early-stage and mid-to-late-stage patients with Parkinson’s disease.

Variable	Early-stage (*n* = 151)	Mid-to-late-stage (*n* = 191)	Test statistic	*p* value
Age, years, median (P25, P75)	66.00(61.00,72.00)	72.00(67.00,75.00)	-5.676^ᵃ^	**<0.001**
Sex, *n* (%)			0.489^ᵇ^	0.485
Male	84(55.6%)	99(51.8%)		
Female	67(44.4%)	92(48.2%)		
Educational level, *n* (%)			3.713^ᵇ^	0.156
Junior high school or below	65(43.0%)	102(53.4%)		
Senior high school/technical secondary school	66(43.7%)	70(36.6%)		
Junior college or above	20(13.2%)	19(9.9%)		
Marital status, n (%)			0.745^ᵇ^	0.388
Married	146(96.7%)	181(94.8%)		
Unmarried/divorced	5(3.3%)	10(5.2%)		
Average monthly household income per capita, *n* (%)			3.715^ᵇ^	0.156
<3,000 CNY	36(23.8%)	55(28.8%)		
3,000–5,000 CNY	55(36.4%)	79(41.4%)		
>5,000 CNY	60(39.7%)	57(29.8%)		
Disease duration, *n* (%)			56.064^ᵇ^	**<0.001**
<5 years	76(50.3%)	34(17.8%)		
5–10 years	62(41.1%)	86(45.0%)		
>10 years	13(8.6%)	71(37.2%)		
Employment status, *n* (%)			5.221^ᵇ^	0.156
Employed	12(7.9%)	5(2.6%)		
Retired	64(42.4%)	87(45.5%)		
Unemployed	43(28.5%)	54(28.3%)		
Farming	32(21.2%)	45(23.6%)		
Comorbid chronic disease, *n* (%)			1.164^ᵇ^	0.281
Yes	87(57.6%)	121(63.4%)		
No	64(42.4%)	70(36.6%)		

Values in bold indicate *p* < 0.05.

### NMSS and PDQ-39 domain scores and between-group comparisons

The median total NMSS score was 34.50 (19.00, 54.00), and the median PDQ-39 total score was 172.92 (113.75, 242.92). Both scores were significantly higher in the mid-to-late-stage group than in the early-stage group: NMSS total score, 41.00 (28.00, 62.00) versus 26.00 (15.00, 43.00); PDQ-39 total score, 192.92 (147.92, 252.92) versus 149.58 (72.08, 190.00) (both *p* < 0.001). Regarding non-motor symptoms, mid-to-late-stage patients scored significantly higher than early-stage patients on all NMSS domains (all *p* < 0.05) except sexual function (NMSS8) and miscellaneous symptoms (NMSS9). Regarding quality of life, mid-to-late-stage patients scored significantly higher than early-stage patients on mobility (PDQ1), activities of daily living (PDQ2), cognition (PDQ6), and communication (PDQ7) (all *p* < 0.05), whereas no significant between-group differences were observed for emotional well-being (PDQ3), stigma (PDQ4), social support (PDQ5), or bodily discomfort (PDQ8) (Table 2).

**Table 2:** Comparison of NMSS and PDQ-39 domain scores among the total sample, early-stage, and mid-to-late-stage groups.

Domain	Total sample (*N* = 342)	Early-stage (*n* = 151)	Mid-to-late-stage (*n* = 191)	*Z value*	*P value*
Non-Motor Symptoms Scale (NMSS)					
Cardiovascular (NMSS1)	2.00(0.00,2.00)	0.00(0.00,2.00)	2.00(0.00,4.00)	−6.15	**<0.001**
Sleep/fatigue (NMSS2)	8.00(4.00,12.00)	6.00(4.00,12.00)	8.00(5.00,12.00)	−3.08	**0.002**
Mood/cognition (NMSS3)	4.00(0.00,16.00)	2.00(0.00,8.00)	8.00(0.00,20.00)	−4.24	**<0.001**
Perceptual problems/hallucinations (NMSS4)	0.00(0.00,2.00)	0.00(0.00,0.00)	0.00(0.00,4.00)	−5.16	**<0.001**
Attention/memory (NMSS5)	2.00(0.00,4.00)	0.00(0.00,4.00)	4.00(0.00,6.00)	−5.01	**<0.001**
Gastrointestinal tract (NMSS6)	6.00(2.00,8.00)	2.00(2.00,6.00)	5.00(3.00,10.00)	−6.16	**<0.001**
Urinary (NMSS7)	4.00(0.00,6.00)	2.00(0.00,6.00)	6.00(0.00,6.00)	−3.75	**<0.001**
Sexual function (NMSS8)	0.00(0.00,1.00)	0.00(0.00,0.00)	0.00(0.00,1.00)	−0.29	0.779
Miscellaneous (NMSS9)	2.00(0.00,6.00)	2.00(0.00,6.00)	3.00(1.00,6.00)	−1.34	0.181
Parkinson’s Disease Questionnaire-39 (PDQ-39)					
Mobility (PDQ1)	27.5(10.00,45.00)	15.00(2.50,35.00)	32.50(22.50,55.00)	−8.60	**<0.001**
Activities of daily living (PDQ2)	41.67(20.83,62.50)	27.50(12.50,50.00)	50.00(25.00,70.83)	−4.11	**<0.001**
Emotional well-being (PDQ3)	12.50(0.00,37.50)	8.33(0.00,33.33)	12.50(0.00,37.50)	−1.46	0.143
Stigma (PDQ4)	6.25(0.00,50.00)	6.25(0.00,50.00)	6.25(0.00,50.00)	−0.30	0.763
Social support (PDQ5)	0.00(0.00,8.33)	0.00(0.00,0.00)	0.00(0.00,16.67)	−1.46	0.144
Cognition (PDQ6)	25.00(12.50,37.50)	18.75(12.50,31.25)	31.25(18.75,43.75)	−6.14	**<0.001**
Communication (PDQ7)	0.00(0.00,16.67)	0.00(0.00,8.33)	8.33(0.00,16.67)	−5.06	**<0.001**
Bodily discomfort (PDQ8)	16.67(0.00,33.33)	16.67(0.00,25.00)	16.67(0.00,33.33)	−1.87	0.061

Values in bold indicate *p* < 0.05.

### Network analysis results

**Figure 1:**
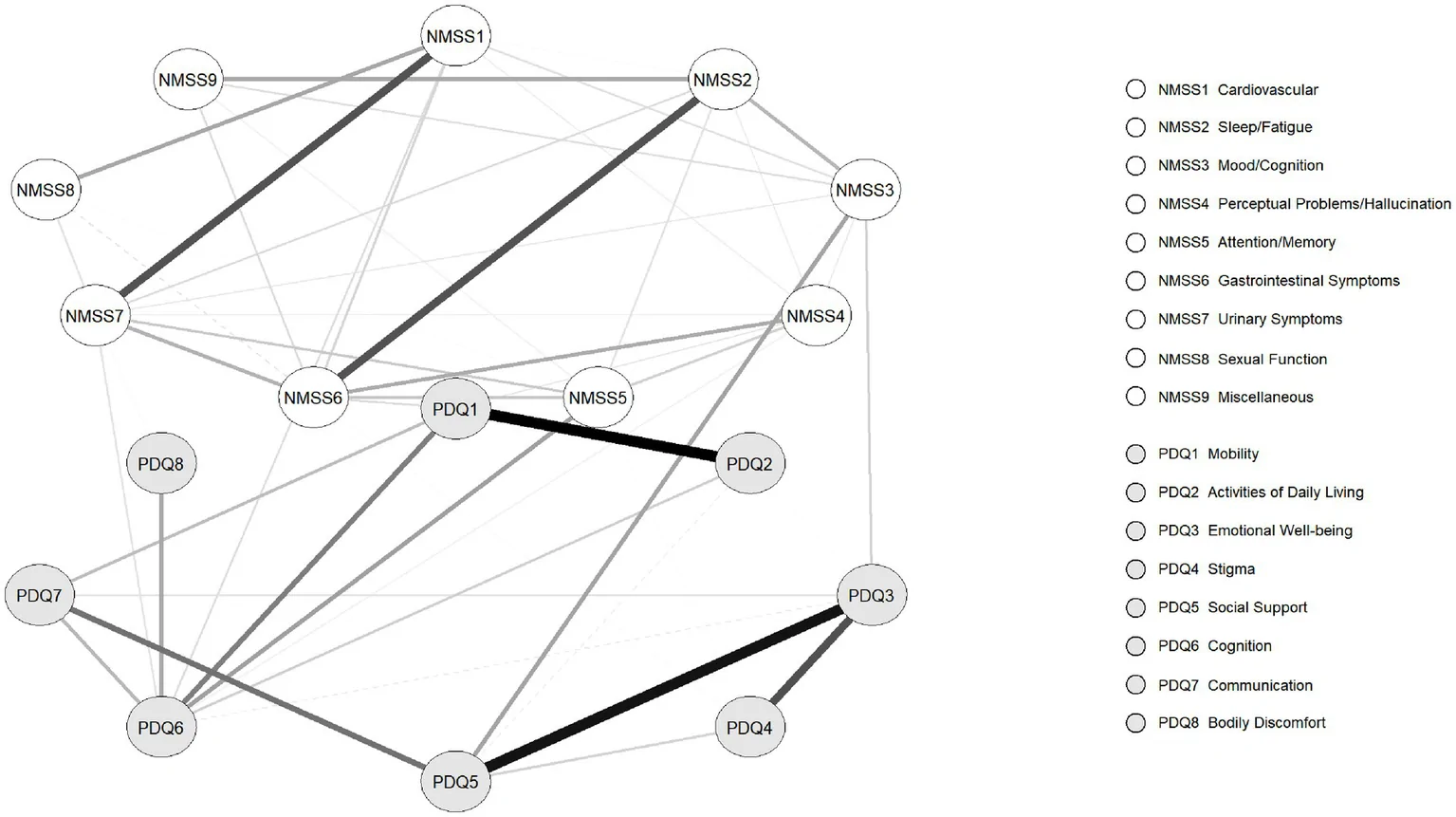
Joint network of NMSS and PDQ-39 in patients with Parkinson’s disease. Edge thickness represents the strength of the correlation between nodes; solid edges indicate positive associations, and dashed edges indicate negative associations.

**Figure 2:**
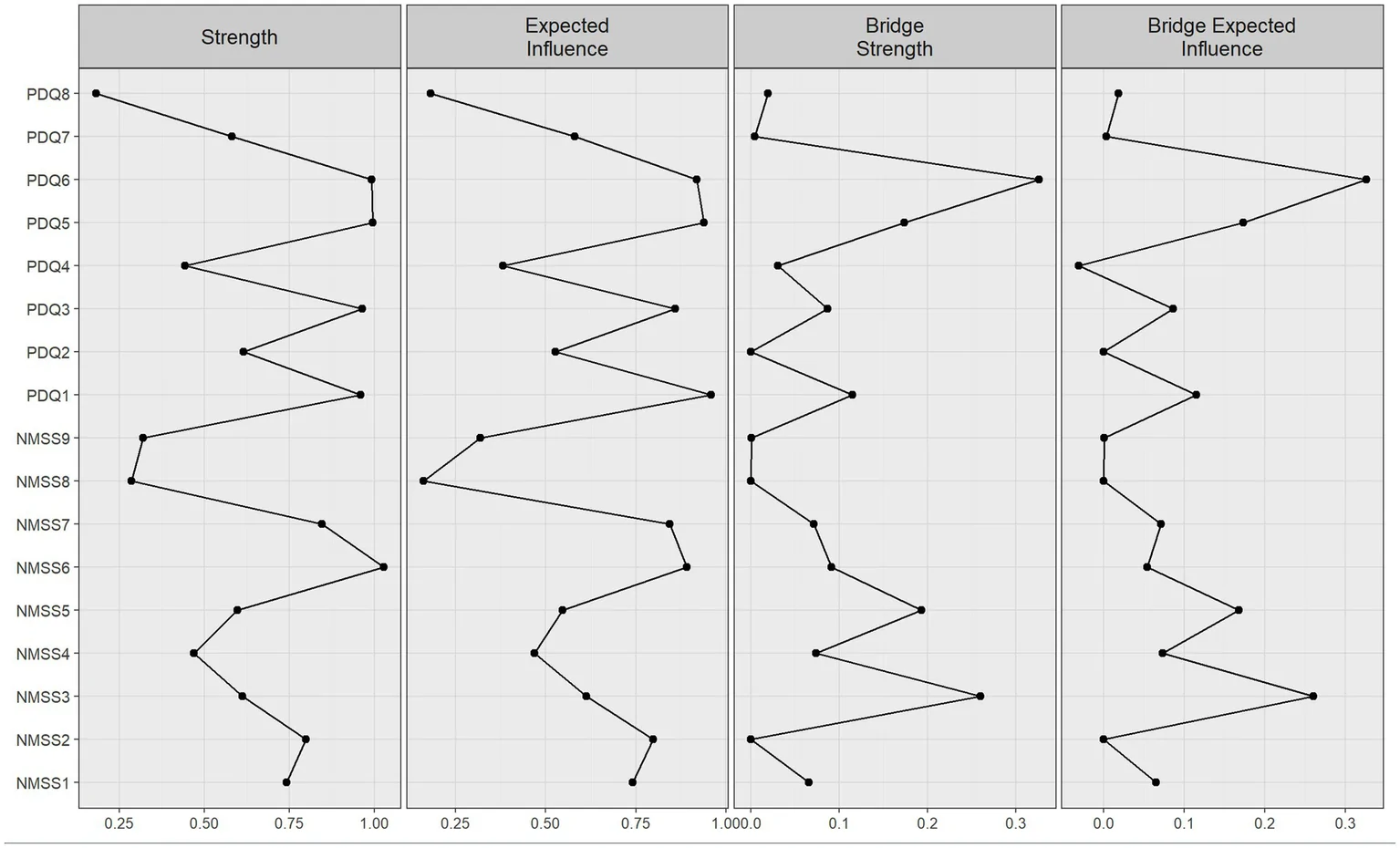
Centrality and bridge centrality indices of nodes in the joint NMSS–PDQ-39 network.

The joint network structure of non-motor symptoms (NMS) and quality of life (QoL) in patients with Parkinson’s disease (PD) is shown in Figure 1, and the centrality indices of network nodes are shown in Figure 2. The overall network density was 0.397. The network comprised 17 nodes, with 54 non-zero edges observed out of 136 possible edges. Among all nodes, NMSS6 gastrointestinal tract symptoms (Str = 1.027), PDQ5 social support (Str = 0.995), and PDQ6 cognition (Str = 0.992) showed the highest strength centrality and were identified as the three core nodes of the network. PDQ6 cognition (Bridge Str = 0.326) and NMSS3 mood/cognition (Bridge Str = 0.260) showed the highest bridge strength among all nodes. PDQ6 cognition (Bridge EI = 0.326), NMSS3 mood/cognition (Bridge EI = 0.260), and PDQ5 social support (Bridge EI = 0.174) showed the highest bridge expected influence. Because all edges crossing the NMS–QoL boundary were positive, bridge expected influence and bridge strength coincided for every node. Regarding expected influence, PDQ1 mobility (EI = 0.960) and PDQ5 social support (EI = 0.939) had the highest values among all nodes.

### Network stability and accuracy

**Figure 3:**
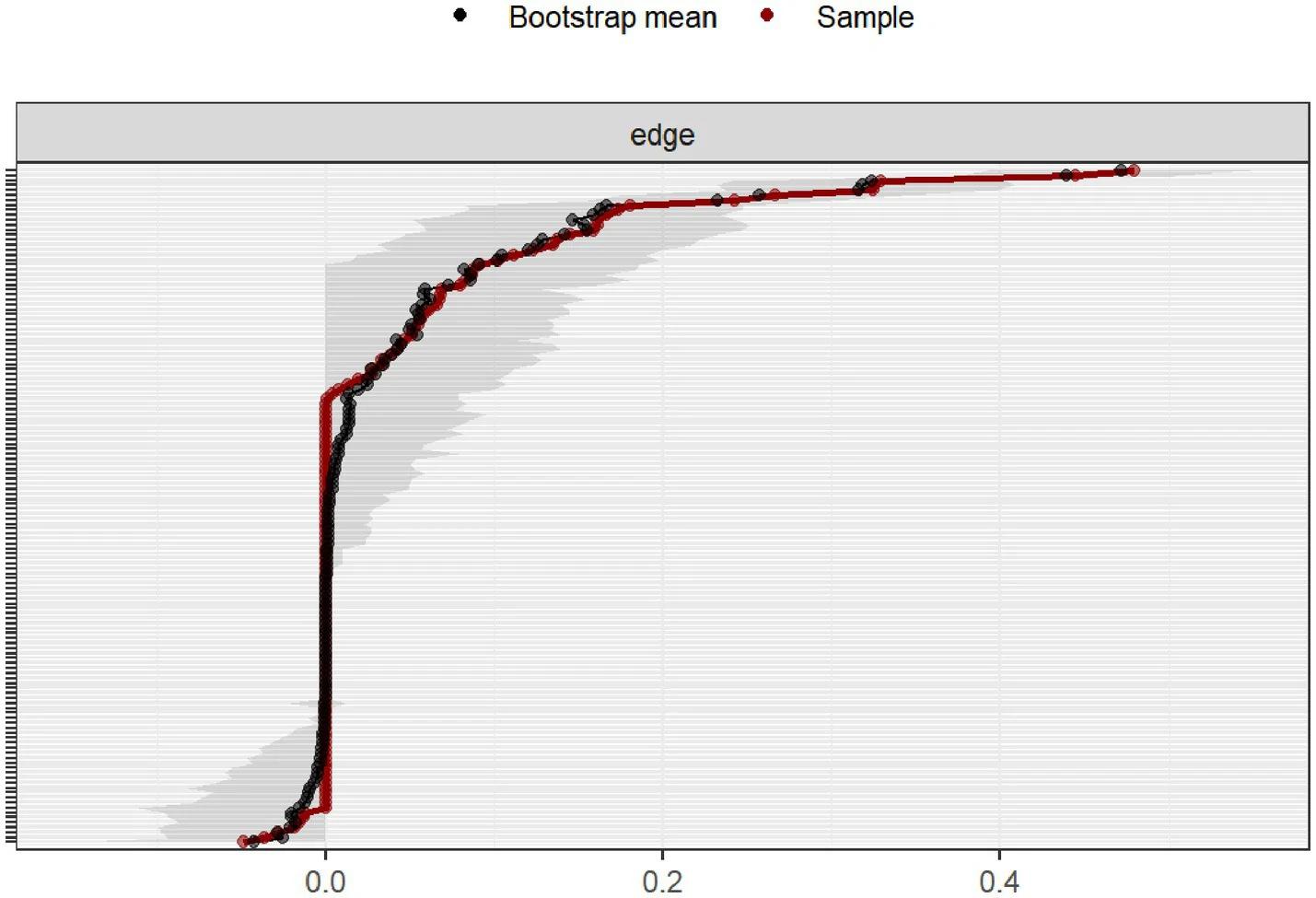
Accuracy of edge-weight estimates (bootstrapped 95% confidence intervals for edge weights).

**Figure 4:**
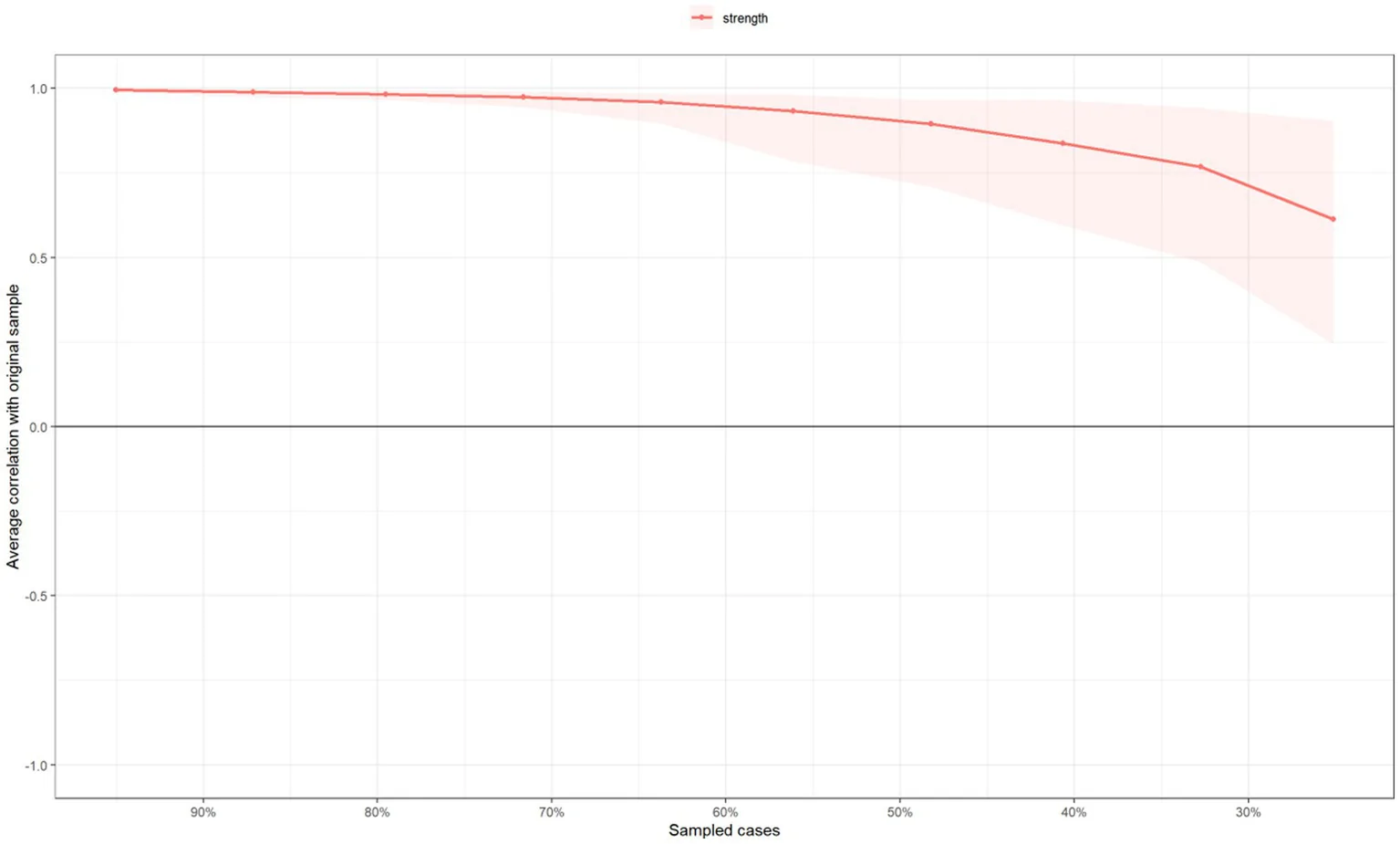
Stability of centrality indices (case-dropping subset bootstrap).

The centrality stability (CS) coefficient for strength centrality of the joint network of non-motor symptoms (NMS) and quality of life (QoL) in patients with Parkinson’s disease (PD) was 0.518, and the CS-coefficients for bridge strength, expected influence, and bridge expected influence were all 0.439, indicating that the network had good stability. The curve representing the observed edge-weight values closely paralleled the curve representing the mean edge-weight values obtained from repeated resampling, indicating high accuracy of edge-weight estimation and confirming that the network had good accuracy (Figures 3, 4).

### Comparison of network structure between early-stage and mid-to-late-stage patients with Parkinson’s disease

**Figure 5:**
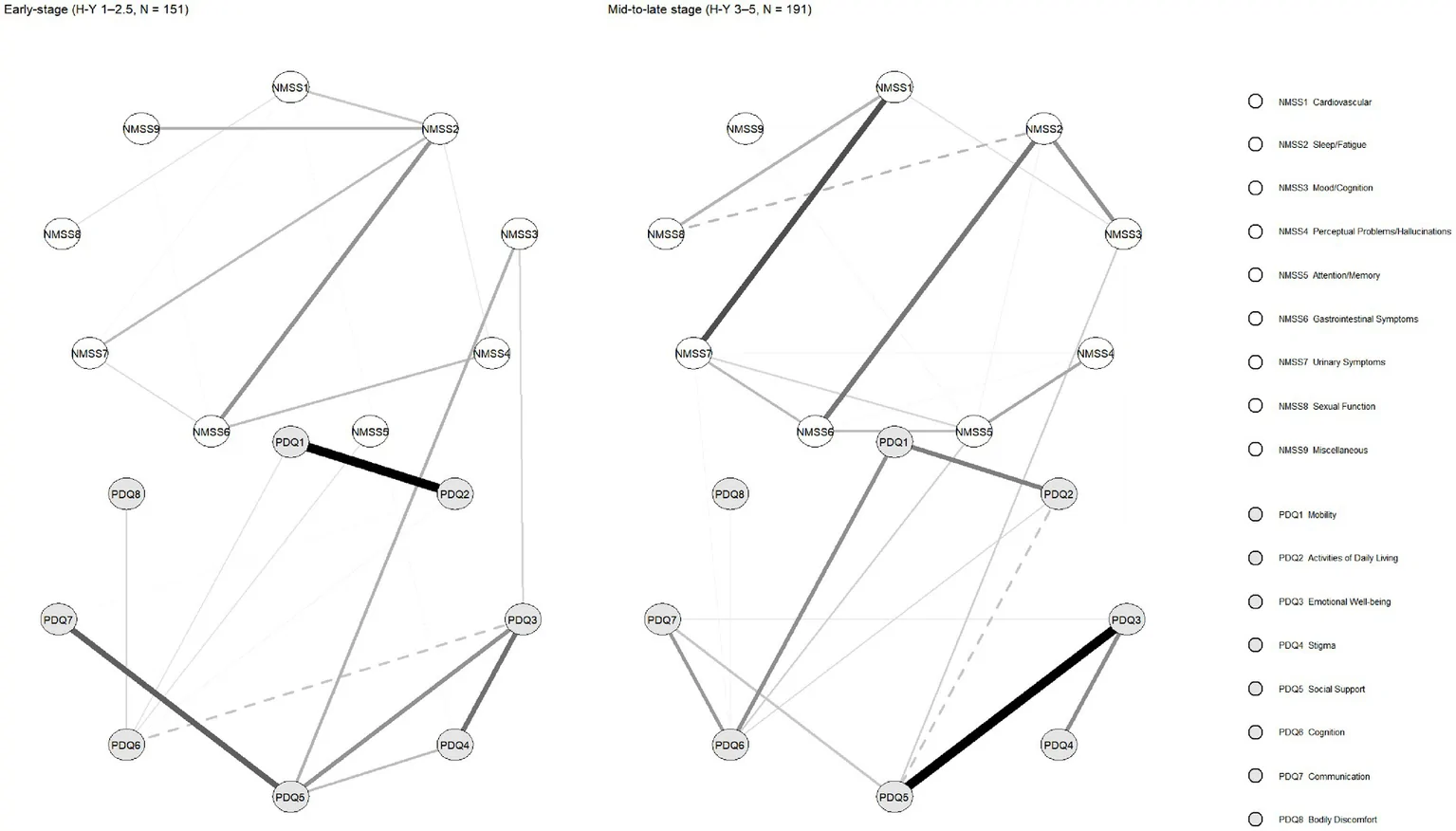
Network analysis of NMSS and PDQ-39 domains in early-stage and mid-to-late-stage patients with Parkinson’s disease. Edge thickness represents the strength of the correlation between nodes; solid edges indicate positive associations, and dashed edges indicate negative associations.

The Network Comparison Test (NCT) showed a statistically significant difference in the overall network structure of the joint NMS-QoL network between early-stage and mid-to-late-stage patients with Parkinson’s disease (M = 0.382, *p* = 0.004). The global network strength was 5.147 in the early-stage group and 3.561 in the mid-to-late-stage group, with no significant difference between groups (S = 1.586, *p* = 0.217). After Benjamini–Hochberg correction for the 136 edge-level comparisons, four edges remained significantly different between the two groups: one within the NMS subnetwork (NMSS2–NMSS8, adjusted *p* = 0.048) and three within the QoL subnetwork (PDQ1–PDQ2, adjusted *p* = 0.048; PDQ2–PDQ5, adjusted *p* = 0.048; and PDQ3–PDQ6, adjusted *p* = 0.014). No cross-scale NMS–QoL edge remained significant after multiple-comparison correction (Figure 5). The early-stage and mid-to-late-stage networks contained 26 and 29 non-zero edges, corresponding to network densities of 0.191 and 0.213, respectively. Case-dropping bootstrap CS coefficients for strength and bridge strength were 0.437 and 0.385 in the early-stage group and 0.383 and 0.256 in the mid-to-late-stage group, respectively, indicating limited stability of the stage-specific centrality estimates.

## Discussion

### Disease-stage-related differences in non-motor symptoms and quality of life among patients with Parkinson’s disease

Both non-motor symptoms (NMS) and quality of life (QoL) burden were considerable among patients with Parkinson’s disease (PD) in this study, and differed significantly across Hoehn-Yahr (H-Y) stages. Regarding NMS, mid-to-late-stage patients scored significantly higher than early-stage patients on seven of the nine NMSS domains (*p* < 0.05), with the exception of sexual function and miscellaneous symptoms, consistent with previous findings (Kumar et al., 2022). The lack of significant between-group differences in sexual function and miscellaneous symptoms may be attributable to the highly heterogeneous nature of sexual dysfunction in PD, which is influenced by multiple factors—including age, medication, and psychological status—rather than being determined solely by disease stage (Ng et al., 2022). Regarding QoL, mid-to-late-stage patients scored significantly higher than early-stage patients on mobility, activities of daily living, cognition, and communication (*p* < 0.05), consistent with the expected trajectory of disease progression (Siderowf et al., 2023). In contrast, no significant between-group differences were observed for emotional well-being, stigma, social support, or bodily discomfort (*p* > 0.05), suggesting that these domains may reflect patients’ psychosocial adaptation to the disease rather than disease severity per se. Previous research has indicated that patients face substantial psychological distress, shifts in social roles, and uncertainty about future disease progression from the time of initial diagnosis; consequently, stigma and the need for social support often emerge early in the disease course and persist throughout its trajectory (Kumar et al., 2022). Taken together, these findings suggest that different dimensions of disease burden in PD follow distinct trajectories: some symptoms intensify progressively with disease advancement, whereas psychosocial factors such as stigma and social support needs may already be present at an early stage and continue to affect QoL over the long term. Clinical nursing practice should therefore not only monitor changes in motor function and NMS over time, but also initiate health education, psychological support, and family caregiving guidance at the time of initial diagnosis, to help patients develop positive coping strategies and improve long-term health outcomes.

### Complex interconnections between NMSS and PDQ-39 domains in patients with Parkinson’s disease

The joint network analysis revealed extensive associations among non-motor symptoms (NMS) and quality of life (QoL) domains in patients with Parkinson’s disease (PD), which together formed a highly interconnected symptom network. Gastrointestinal tract symptoms (NMSS6), social support (PDQ5), and cognition (PDQ6) were identified as the three core nodes of this network. Nodes with high strength centrality are those most strongly connected with multiple other domains after conditioning on all remaining domains (Yang et al., 2022). Gastrointestinal tract symptoms showed the highest strength centrality in the joint network. Previous studies have shown that gastrointestinal dysfunction is among the most common and earliest-appearing NMS in PD, often preceding motor symptoms by several years, and that it affects nutrient intake, drug absorption, and the therapeutic efficacy of levodopa-based medications (Sharma et al., 2025; Lubomski et al., 2020). *This centrality likely reflects the broad physiological reach of gastrointestinal dysfunction in PD, though, given the cross-sectional design, it reflects association rather than a causal or driving role*. Gastrointestinal dysfunction may also sustain neuroinflammation and abnormal α-synuclein aggregation via the gut-brain axis, thereby further accelerating disease progression. Because gastrointestinal symptoms are also closely related to sleep, mood, and autonomic function, they are more likely to form extensive connections throughout the network. A similar pattern was reported at the item level by Heimrich et al. (2023b), who found non-motor items across several domains most strongly linked to HRQoL in the COPPADIS cohort, suggesting this interconnected structure is not unique to gastrointestinal symptoms. Although social support did not differ significantly between H-Y stages in this study, its high strength centrality suggests that social support remains closely linked to multiple NMS and QoL domains and serves as an important influencing factor throughout the entire disease course. As the disease progresses, patients become increasingly dependent on family and social support; adequate social support can improve treatment adherence, self-management ability, and psychological adaptation, whereas insufficient social support may lead to social isolation and a heightened sense of stigma, further reducing QoL (Crooks et al., 2025). Cognition (PDQ6) exhibited not only high strength centrality but also the highest bridge strength among all nodes; mood/cognition (NMSS3) likewise showed high bridge strength, suggesting that cognition-related factors serve both as a core dimension within the QoL network and as an important hub linking NMS to QoL. Previous studies have shown that cognitive decline is an important determinant of QoL in patients with PD, with greater impairment associated with poorer QoL (Bock et al., 2022). Although both domains relate to cognition, the cognition dimension of the PDQ-39 primarily reflects the impact of cognitive decline on daily functioning (Santos-García et al., 2023),whereas the mood/cognition dimension of the NMSS focuses on symptoms such as anxiety, depression, apathy, and impaired attention (Camerucci et al., 2024). These two dimensions thus capture cognition-related problems from two distinct perspectives—functional outcome and symptom presentation—and may interact bidirectionally with declining QoL to form a vicious cycle involving emotional disturbance and cognitive impairment (Munzesheimer et al., 2025). However, this conceptual distinction is not matched by complete separation at the item level, and the same applies to the mood- and function-related domains of the two instruments. Part of the observed cross-scale connectivity may therefore reflect shared item content, and the bridge findings are best read as identifying the domains at which the symptom-based and quality-of-life-based descriptions of the disease converge most strongly. Regarding expected influence, mobility (PDQ1) and social support (PDQ5) showed the highest values among all nodes, indicating that these two nodes showed the greatest net positive connectivity within the estimated network (Yang et al., 2022). These expected-influence findings, together with the strength and bridge results described above, highlight the complementary value of a domain-level approach. Previous studies focused mainly on item-level or single-scale networks (Heimrich et al., 2023b; Heimrich et al., 2023a; Schönenberg et al., 2023), while Deng et al. examined a symptom network in early-to-mid-stage PD (Deng et al., 2025). By modeling all nine NMSS and eight PDQ-39 domains within one network, the present analysis summarizes how broad NMS and QoL domains are conditionally connected. Taken together, gastrointestinal tract symptoms, cognition, mobility, and social support demonstrated high centrality and/or bridge strength in the present joint network. As this network is cross-sectional and undirected, this indicates that these domains are the most densely interconnected within the network, rather than that they play a causal role in maintaining network stability or in the transmission of NMS to QoL. These domains may therefore be considered relevant associated domains for nursing assessment and possible targets for future longitudinal and intervention studies.

### Heterogeneity of the Joint Network Across Hoehn-Yahr Stages in Patients with Parkinson’s Disease

The Network Comparison Test (NCT) was used to examine differences in the joint network between patients with Parkinson’s disease (PD) at different Hoehn-Yahr (H-Y) stages. The results showed that the overall network structure differed significantly between early-stage and mid-to-late-stage patients, whereas global network strength did not differ significantly, with changes confined to a subset of key edges. Because the early-stage and mid-to-late-stage groups differed significantly in age and disease duration, the stage-based network comparison should be interpreted primarily as an exploratory analysis of possible differences in association patterns across disease stages. These findings suggest that the pattern of association between non-motor symptoms (NMS) and quality of life (QoL) may differ across H-Y stages, rather than simply reflecting an increase in the severity of individual symptoms. After correction for multiple comparisons, only four of the 136 tested edges showed statistically significant between-group differences. These comprised one edge within the NMS subnetwork, linking sleep/fatigue and sexual function, and three edges within the QoL subnetwork, linking mobility with activities of daily living, activities of daily living with social support, and emotional well-being with cognition. No cross-scale NMS–QoL edge remained significantly different after correction. Therefore, the corrected edge-level findings do not support widespread stage-related differences in cross-scale NMS–QoL connectivity. Rather, in the present sample, detectable between-group differences were concentrated within the NMS and QoL subnetworks. Given the number of comparisons, the subgroup sample sizes, and the differences in age and disease duration between groups, these local findings should be interpreted as exploratory and hypothesis-generating indications of possible differences in association patterns, rather than as definitive evidence of disease-stage-specific symptom pathways.

## Limitations

This study has several limitations. First, the sample was recruited by convenience sampling from a single hospital and the design was cross-sectional; the findings may therefore not be representative or generalizable, no causal or longitudinal inferences can be drawn, and the undirected network and its centrality indices should be interpreted descriptively rather than as directional effects. Second, the stage-stratified comparison should be considered exploratory, because the groups differed significantly in age and disease duration, which were not adjusted for in the network comparison, and only four of the 136 tested edges remained significant after multiple-comparison correction. Third, all measures were self-reported and thus susceptible to recall and social desirability biases, and content overlap between some NMSS and PDQ-39 domains may have contributed to the observed bridge centrality. Fourth, motor severity, medication profiles and treatment state, motor complications, PD subtype, caregiver support, and standardized cognitive screening were not included in the network, leaving potential residual confounding. Future multicenter, large-sample longitudinal studies are needed to clarify the dynamic evolution of the relationship between NMS and QoL in patients with PD; nursing practice should therefore implement stage-specific, precision care strategies tailored to the network characteristics of each disease stage.

## Conclusion

This study found that gastrointestinal tract symptoms, social support, and cognition were the core nodes of the joint network, with cognition and mood/cognition showing high bridge strength, and mobility and social support showing high expected influence. As this network is cross-sectional and undirected, these findings indicate that the above NMS and QoL domains are the most densely interconnected within the network, not that they causally drive it. These domains may therefore be considered relevant associated domains for nursing assessment and possible targets for future longitudinal and intervention studies. Network comparison analysis showed that the overall network structure differed significantly between early-stage and mid-to-late-stage patients with Parkinson’s disease (PD), whereas global network strength did not differ significantly, indicating that the pattern of symptom associations—but not the overall strength of these associations—may differ across Hoehn-Yahr (H-Y) stages. Nursing practice may therefore consider stage-specific care strategies tailored to the network characteristics of each disease stage.

## Data Availability

The raw data supporting the conclusions of this article will be made available by the authors, without undue reservation.
